# Endovascular repair with covered stent for a giant hepatic artery pseudoaneurysm: a case report and literature review

**DOI:** 10.3389/fmed.2025.1579860

**Published:** 2025-05-02

**Authors:** Qingchun Hou, Wei Wei, Weiming Wang, Weijian Mao, Yanneng Xu, Wei Hu, Guangyan Si, Gang Yuan

**Affiliations:** ^1^Department of Vascular Surgery, Zigong Fourth People's Hospital, Zigong, Sichuan, China; ^2^Department of General Surgery, The People's Hospital of Longmatan District, Luzhou, Sichuan, China; ^3^Department of General Surgery (Vascular Surgery), The Affiliated Hospital of Southwest Medical University, Luzhou, China; ^4^Department of Intervention and Vascular, The Affiliated Traditional Chinese Medicine Hospital, Southwest Medical University, Luzhou, China

**Keywords:** hepatic artery pseudoaneurysm, endovascular therapy, cover stent, transcatheter arterial embolization, case report

## Abstract

Hepatic artery pseudoaneurysm (HAP), a rare life-threatening complication, typically occurs following trauma or surgical procedures such as liver transplantation. Initially asymptomatic, its rupture risk escalates with increasing size. Once ruptured, it carries an extremely high mortality rate, and delayed intervention may lead to fatal hemorrhagic shock. Consequently, early diagnosis and timely intervention are pivotal in managing HAP. Herein, we present a case of a HAP measuring approximately 10 cm in diameter. The patient was admitted with abdominal pain, and the clinical history did not indicate a definitive etiology. Computed tomography angiography revealed that the rupture site of the pseudoaneurysm was located in the common hepatic artery, with partial thrombus formation within the aneurysmal sac. The expansive lesion compressed the hepatic artery, resulting in hypoperfusion. Following multidisciplinary consultation and obtaining informed consent from the patient and her family, the patient underwent endovascular treatment under local anesthesia. During the procedure, two covered stents were successfully implanted. Postoperatively, the rupture of the HAP was effectively excluded, hepatic arterial patency was restored, and the patient’s abdominal pain was alleviated significantly. She was discharged 5 days after receiving antiplatelet and anti-infective therapy. Long-term antiplatelet treatment was continued, and at a one-year follow-up, the stent remained patent with no evidence of lesion recurrence. This case report, combined with literature review, aims to analyze HAP etiology and summarize diagnostic and therapeutic experiences.

## 1 Introduction

Hepatic artery pseudoaneurysm (HAP) is a pulsatile hematoma-like lesion formed by extravasated blood following damage to the hepatic arterial wall, with the hematoma encapsulated by organized fibrous tissue. It commonly arises secondary to hepatic trauma, abdominal surgery, liver transplantation, or biliary infections. In the early stages, symptoms may not be apparent due to the small size of the lesion. However, as the lesion progresses, symptoms such as abdominal distension and pain may occur. Once it ruptures and bleeds, the mortality rate can be as high as 60–70%, necessitating immediate intervention upon diagnosis (1, 2).

The inducing factors of HAP primarily includes trauma, infection, and vascular degeneration. Hepatic artery injury from traumatic or iatrogenic injuries is common such as abdominal surgery, interventional catheterization, or orthotopic liver transplantation, etc. Moreover, the intraperitoneal infectious diseases such as pancreatitis, pyogenic cholangitis, or hepatic abscesses may also cause vascular erosion, thus inducing HAP formation. Furthermore, vascular degenerative diseases, such as atherosclerosis, penetrating ulcers, or autoimmune diseases causing vascular deterioration, are also significant contributing factors that accelerate the formation of HAP (2–6).Currently, with the widespread adoption of laparoscopic techniques and imaging examination, more and more HAPs are diagnosed clinically, but there is still a lack of unified standards for their treatment. Historically, surgical repair dominated management. Recently, with the development of interventional radiology, in addition to surgical repair, choosing a more minimally invasive endovascular treatment, such as covered stent implantation or selective hepatic artery catheterization embolization (TAE), seems to have become a new and more popular method (1, 6, 7). TAE is preferred for distal branch lesions, whereas preservation of hepatic arterial flow is critical for main trunk or major branch pseudoaneurysm to avoid hepatic ischemia, infarction, abscesses, or liver failure. In such cases, covered stent implantation offers a viable alternative (6). Clinical studies have validated the efficacy and safety of stent grafts for HAPs (8, 9). However, most of the literature reports that the size of pseudoaneurysms is still small, and HAP with a diameter exceeding 5 cm is rare, while giant pseudoaneurysms with a lesion diameter exceeding 8 cm have not been reported. Here, we describe the endovascular management process of a giant HAP measuring nearly 10 cm in diameter, and by reviewing relevant literature, analyze and summarize the diagnosis and treatment experience of HAP, aims to refine diagnostic and therapeutic strategies for HAP, particularly advocating for endovascular interventions in managing such rare, life-threatening entities.

## 2 Case presentation

A 67-year-old female was admitted with epigastric pain persisting for over 1 week. The pain began insidiously without identifiable triggers, characterized by intermittent distending discomfort lasting minutes to hours, tolerable in severity. It was accompanied by lower abdominal needle-like pain. Notably, the patient denied nausea, vomiting, chest tightness, chest pain, cough, sputum production, abdominal distension, diarrhea, hematochezia, or hematuria. A computed tomography examination in the local hospital identified a huge space-occupying lesion in the liver-stomach gap, suspected to be a pseudoaneurysm, which may have originated from the distal common hepatic artery. The patient was transferred urgently due to the high risk of rupture. Her medical history was unremarkable aside from childhood right eye blindness (etiology unknown) and a prior excision of a knee skin mass over a decade ago. She denied significant comorbidities or previous trauma.

**Figure 1:**
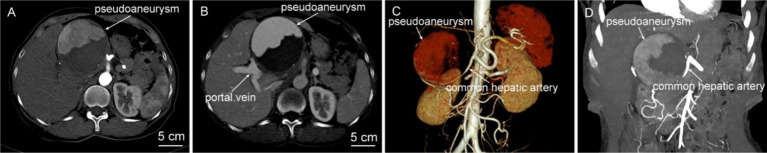
Abdominal CTA examination. **(A,B)** Axial CT images during the arterial phase and portal venous phase show a spherical occupying lesion in the upper abdomen, measuring approximately 8.5 × 7.8 × 8.0 cm. There is partial enhancement within it, accompanied by a large amount of thrombus formation. **(C,D)** CTA reconstructions reveal the common hepatic artery is compressed and narrowed, and the branches of the superior mesenteric artery—pancreaticoduodenal artery and the right hepatic artery are open. The right hepatic artery is mainly supplied by the pancreaticoduodenal artery, and the left hepatic artery originates from the left gastric artery.

Physical examination: A pulsatile, border-cleared, texture-toughed mass (~10 × 10 cm) was palpated in the right upper abdomen, tender on pressure, with pulsations synchronous with the cardiac cycle. In addition, a blowing-like vascular murmur can be heard in the abdominal wall. Laboratory examination: Hemoglobin: 105 g/L; high-sensitivity C-reactive protein: 25.55 mg/L; D-dimer: 3.26 mg/L. Liver and renal function tests were unremarkable. Computed tomography angiography (CTA) showed that there was a spheroid shadow about 8.5 × 7.8 × 8.0 cm in size below the left lobe of the liver in the hepatic hilum area. The contrast agent could be seen entering, and part of the lesion was enhanced, with a large amount of thrombosis formation, which was consistent with the manifestations of pseudoaneurysm, suggesting the possibility of a common hepatic artery pseudoaneurysm. The hepatic artery was compressed and narrowed, and the rupture site of the pseudoaneurysm was not clearly displayed. Collateral circulation via the superior mesenteric-pancreaticoduodenal artery and right hepatic artery, with the right hepatic artery primarily supplied by the pancreaticoduodenal artery and the left hepatic artery originating from the left gastric artery. The main trunk of the portal vein was compressed and narrowed with collateral branches open, and the common bile duct was compressed with mild dilation of the intrahepatic bile ducts (Figure 1).

**Figure 2:**
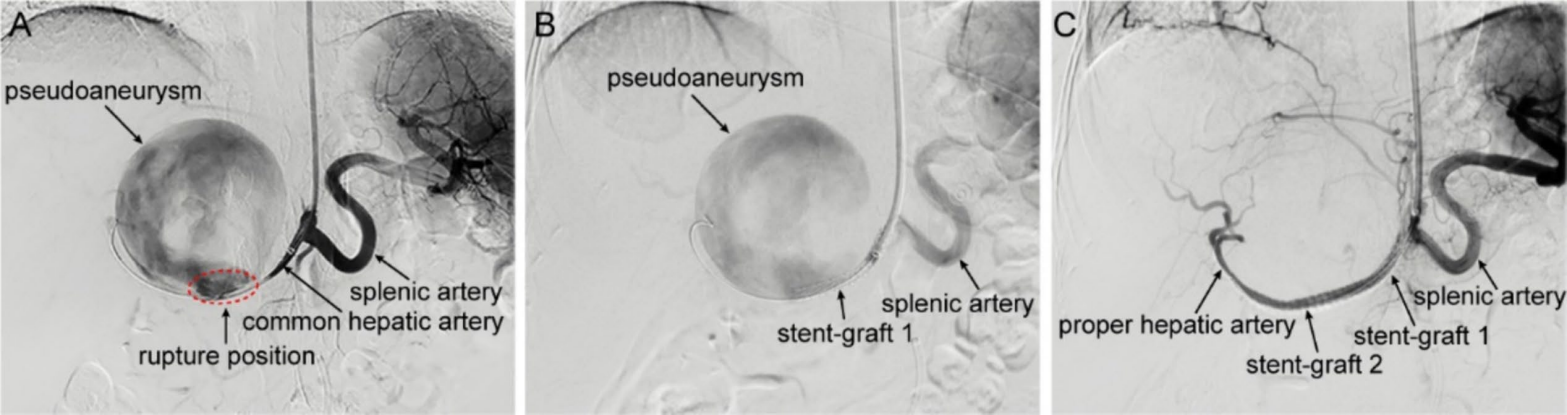
Intraoperative DSA imaging. **(A)** Hepatic arteriography shows contrast agent overflowing from the common hepatic artery, presenting as a spheroid aneurysmal dilation with a size of about 9 × 9 cm, on the verge of rupture. The common hepatic artery is significantly narrowed due to compression, and the distal proper hepatic artery and its branch vessels are not visible. **(B)** After the implantation of a covered stent-graft in the common hepatic artery, the pseudoaneurysm is still visible on angiography, and the distal hepatic artery remains invisible. **(C)** After the implantation of a second stent-graft in the hepatic artery, the pseudoaneurysm disappears on angiography, and the distal proper hepatic artery and its branch vessels are clearly visible.

Following multi-disciplinary consultation and informed consent, the patient underwent local anesthesia-assisted hepatic artery angiography and endovascular repair. Initial digital subtraction angiography (DSA) confirmed a spherical pseudoaneurysm (~9 × 9 cm) arising from the mid-segment of the common hepatic artery, with active contrast extravasation at the rupture site (Figure 2A). During the operation, a 6 mm × 58 mm balloon-expandable covered stent (Bard, United States) was implanted into the common hepatic artery. However, contrast agent extravasation was still visible on angiography (Figure 2B), suggesting incomplete coverage of the rupture site. To address this, another 5 mm × 37 mm balloon-expandable covered stent (Bard, United States) was implanted to bridge with the first stent. After re-angiography, the rupture of the pseudoaneurysm was completely isolated, the stent-grafts remained patent, without contrast agent extravasation, and the common hepatic artery and its distal proper hepatic artery were well visualized, indicating a successful operation (Figure 2C).

**Figure 3:**
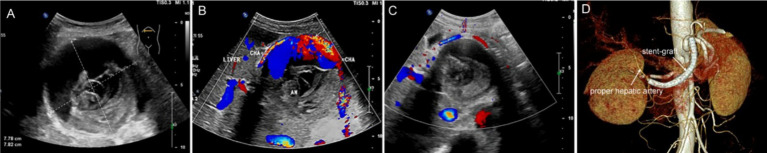
Imaging evaluation after interventional operation. **(A)** Preoperative ultrasound shows a spherical mass in the upper abdomen with clear boundaries, measuring approximately 8 × 8 cm, with uneven internal echo. **(B)** Doppler imaging shows a large amount of blood flow signals within the lesion connected to the common hepatic artery. **(C)** Three months after the operation, ultrasound review shows the lesion has decreased in size, with more uniform internal echo and no obvious blood flow signals. **(D)** One year later, CTA shows the stent graft is patent, with patent proper hepatic artery and its distal branches as well.

The patient’s abdominal pain improved significantly postoperatively, and she was successfully discharged after 5 days of antiplatelet and anti-infective therapy. After discharge, the patient continued to receive dual antiplatelet therapy with low-dose aspirin (100 mg/d) and clopidogrel (75 mg/d) for 6 months, and then the treatment was adjusted to single antiplatelet therapy with low-dose aspirin (100 mg/d). Three months later, the follow-up ultrasound showed that the volume of the lesion was significantly reduced and the lesion diameter was also reduced by nearly 2 cm compared with that before operation, no obvious blood flow signal was seen in the lesion site, and a large amount of thrombosis formation was visible (Figures 3A−C). One year later, the follow-up abdominal CTA showed that the stent-grafts were unobstructed, and the distal proper hepatic artery and its branches were still well visualized (Figure 3D).

## 3 Discussion

HAP is clinically rare, with an incidence of approximately 0.27–2%, and carries a rupture risk of 21–80% (3, 10). Despite its rarity, it is recognized as a severe and potentially fatal complication, with mortality rates as high as 69–75% (3, 11, 12). Therefore, prompt intervention is required once a definitive diagnosis is established. The pathogenesis and exact etiology of HAP remain unclear. Some studies classify HAP into two distinct types: intrahepatic and extrahepatic, based on associated predisposing factors (13). Intrahepatic HAP is relatively uncommon and is often caused by iatrogenic injuries, including percutaneous transhepatic procedures such as liver mass biopsy, cholangiography, biliary stent implantation, and hepatic artery chemoembolization (13, 14). In contrast, extrahepatic pseudoaneurysms are more prevalent, accounting for over 69% of HAP cases (13, 15). Local infection and trauma are the two primary predisposing factors, associated with both types of pseudoaneurysms but demonstrating a stronger correlation with the extrahepatic type (3, 10, 16). Studies indicate that approximately 80% of HAP cases are infection-related, where bacteria erode the arterial adventitia, promoting pseudoaneurysm formation (5, 16). For instance, infected bile due to bile leakage is believed to directly weaken and erode vascular walls, increasing HAP risk (17). Infections may also accelerate bile duct wall thinning and HAP rupture into the biliary tract or duodenum, triggering biliary or gastrointestinal hemorrhage. Additionally, trauma or intraoperative injury is a significant predisposing factor for extrahepatic HAP (4). This is usually triggered by improper operation during surgery, such as mistakes in dissection during cholecystectomy or indirect induction of HAP caused by thermal damage to blood vessels due to heat radiation generated during the use of electrocautery or ultrasonic scalpel (18).

Although HAP is life-threatening, it is often difficult to detect before rupture because of atypical early symptoms. Upon HAP rupture, intra-abdominal hemorrhage may manifest as peritoneal signs (e.g., abdominal tenderness) or localized hematoma in the hepatogastric space causing epigastric distension and pain (19). Some patients may also present with gastrointestinal bleeding due to bleeding into the gastrointestinal tract (16). Literature also reports HAP involvement in the biliary system, leading to hepatic artery-biliary fistulas and subsequent haemobilia (20). Given the high post-rupture mortality, early diagnosis and timely intervention before rupture are critical for favorable outcomes.

The diagnosis of HAP relies on the patient’s medical history (particularly prior trauma, surgical history, or abdominal infections) and is confirmed through imaging modalities such as ultrasound, contrast-enhanced CT, and/or DSA (4, 5). Although selective hepatic arteriography is an invasive diagnostic method, it remains the most accurate modality for HAP diagnosis. During the procedure, it visualizes damaged vascular walls and contrast extravasation, precisely identifying the lesion’s location, thus earning its reputation as the gold standard for HAP diagnosis (21). This patient had no history of abdominal trauma, surgery, infection, or vascular disease. Upon discovery, the HAP was notably large, with intermittent epigastric pain and discomfort as the primary symptom. Relevant laboratory examinations did not suggest signs of intra-abdominal infection. Based on clinical presentation, the case was presumed to be an idiopathic hepatic artery pseudoaneurysm of unknown etiology. Selective hepatic arteriography accurately identified a vascular wall defect and contrast extravasation in the common hepatic artery, clearly delineating the pseudoaneurysm’s morphology, size, and the precise location of the rupture. These findings provided robust support for formulating the treatment plan.

The traditional treatment methods for HAP are surgical operations (partial hepatic lobectomy or arterial ligation), which can achieve the purpose of hemostasis. However, these methods are highly invasive, with a high incidence of complications and a slow recovery. Consequently, they are no longer considered the first-line treatment. In recent years, with advancements in endovascular techniques, the treatment of HAP mainly adopts more minimally invasive endovascular methods, such as TAE or stent-graft implantation, with surgery reserved only for exceptional cases (22). In most reported cases, TAE appears to be the optimal approach for managing HAP. However, simple vascular embolization may cause occlusion of the main hepatic artery trunk, potentially leading to acute liver failure, making it unsuitable for embolization of lesions in the main hepatic artery trunk (23, 24). Therefore, it is necessary to evaluate sufficient arterial and collateral blood flow before hepatic artery embolization, so as to reduce the risk of local ischemia and prevent complications related to reduced blood flow. Endovascular stent repair is a procedure that uses a covered stent to completely isolate the rupture of the diseased artery from the HAP, reducing the pressure within the aneurysmal cavity and stopping blood flow, ultimately leading to thrombus formation within the cavity. By maintaining hepatic arterial patency through the stent’s radial support, this technique prevents hepatic tissue ischemia and serves as an effective endovascular treatment for HAP (7). Pedersol et al. reported their experience with endovascular treatment in 30 HAP patients, where 25 underwent covered stent placement and 5 received coil embolization. The technical success rate for stent implantation was 92%, with hepatic arterial patency achieved in 88% of cases. Short-term follow-up revealed a high stent patency rate (81%), which declined to 40% in mid- to long-term follow-up. Although these data suggest that the long-term patency rate of stents may not be optimistic, the number of cases included in the study was too small, and some patients were lost to follow-up, making it impossible to accurately assess the long-term patency of the stents (1). Other studies have reported widely varying stent patency rates, ranging from 42 to 100% (8, 9, 25, 26). This discrepancy may relate to variability in postoperative antithrombotic regimens. Reported anticoagulation and antiplatelet protocols after stent implantation vary significantly across studies, ranging from no therapy to lifelong aspirin (150 mg/d) combined with clopidogrel (75 mg/d for 12 weeks) (8, 27).

The optimal use of anticoagulation or antiplatelet therapy following stent implantation remains controversial. Typically, anticoagulation and antiplatelet therapy are required for at least 3–6 months after the implantation of a covered stent to avoid thrombosis within the stent. However, for patients with contraindications to anticoagulation, the use of anticoagulants or antiplatelet agents carries a risk of recurrent bleeding. For this reason, some researchers believe that a larger-sized covered stent can be selected to reduce the requirements for postoperative anticoagulation and antiplatelet therapy (8). According to guidelines from the European Society of Cardiology and the Society for Vascular Surgery, there is no specific recommendation for single or dual antiplatelet therapy after endovascular treatment of visceral arteries. However, empirically, most medical centers prescribe daily clopidogrel (75 mg) combined with low-dose aspirin for durations ranging from 1 month to 1 year (28). Although a direct causal link between stent occlusion and discontinuation of dual antiplatelet therapy has not been definitively proven, the concurrent occurrence of stent occlusion and cessation of antiplatelet therapy suggests that except in patients at high risk of bleeding dual antiplatelet therapy may offer potential benefits for maintaining long-term stent patency (29). Reassuringly, during long-term follow-up, while some patients experienced stent occlusion, 86% of these individuals developed collateral circulation in the hepatic artery, effectively preserving hepatic blood supply without ischemic symptoms. Only 14% of patients developed hepatic abscesses, potentially triggered by acute ischemia (1).

In previous reports, HAP with diameters exceeding 5 cm have been extremely rare. Here, by reviewing relevant literature in recent years, we have summarized the treatment methods and clinical outcomes of previously reported HAP cases ≥5 cm in Table 1 below. Although treatment approaches are diverse, including open surgical repair, TAE, and stent implantation, it is evident that for patients with giant HAP, endovascular therapy demonstrates significantly superior prognosis compared to open surgery (30–39). In this case, the patient presented with a large HAP, significant stenosis due to hepatic artery compression, and no visualization of distal vasculature. Although collateral communication existed between the left hepatic artery and the left gastric artery, the HAP rupture site was located in the common hepatic artery. Direct embolization of the common hepatic artery would inevitably lead to extensive hepatic ischemia, posing a high risk of postoperative liver failure. Therefore, simple embolization was deemed unsuitable for this patient. The use of a covered stent not only effectively sealed the HAP but also preserved hepatic arterial perfusion. Although the origin of the gastroduodenal artery was also covered by the stent, its extensive collateral circulation with the superior mesenteric artery alleviated concerns about ischemic complications. Consequently, we successfully treated this giant common HAP using covered stent endovascular repair. Postoperatively, the patient was assessed to have a low bleeding risk and thus received dual antiplatelet therapy. At the 1-year follow-up, the stent remained patent, liver function was normal, and the patient exhibited a favorable prognosis. While covered stent placement effectively sealed the rupture site of pseudoaneurysm and controlled bleeding, postoperative infection prevention remained critical due to the risk of secondary infection from post-hemorrhagic hematoma. In this case, although preoperative routine blood tests did not indicate obvious signs of infection, we still carried out prophylactic anti-infection treatment. The patient had a good prognosis after the operation, without fever, chills or other infectious complications.

**Table 1:** The HAP cases ≥5 cm with pathogenesis, management approaches and outcomes reported previously.

Size of HAP	Pathogenesis	Management approaches	Outcomes	References
8.5 × 10.2 cm	Unclear	Surgical treatment by laparotomy	Success, alive	(30)
8.0 × 6.5 cm	Liver transplantation	Stent implantation is to be performed	Preoperative death	(10)
Diameter 6.5 cm	Post gunshot injury	Balloon assisted thrombin injection	Success, alive	(31)
14.8 × 9.8 cm	Unclear	Open surgical repair	Success, alive	(32)
5.8 × 4.1 × 3.0 cm	Abdominal trauma	TAE with gel foam, particle, and microcoils	Success, alive	(33)
6.7 × 6.0 × 5.6 cm	Unclear	The patient requested conservative treatment	Died 5 days after admission	(34)
10.3 × 8.5 × 8.1 cm	Post radical gastrectomy	Endoaortic stent grafting	Success, alive 5 years	(35)
Diameter 14.0 cm	Unclear	TAE with coils	Success, alive	(36)
4.5 × 5.7 cm	Chronic pancreatitis	TAE with coils	Success, alive 1 years	(37)
8.0 × 7.2 × 7.0 cm	Chronic pancreatitis	Aneurysm resection and common hepatic artery bypass	Died 135 days after surgery	(38)
Diameter 5.9 cm	Unclear	TAE with coils	Success, alive 3 years	(39)

## 4 Conclusion

The etiology and mechanisms underlying HAP remain unclear, and their nonspecific clinical presentation complicates early diagnosis. Therefore, imaging screening should be emphasized for patients with a history of hepatic trauma, surgery (particularly liver transplantation or biliary-enteric anastomosis), or intra-abdominal infections. Treatment should adhere to personalized principles, with the choice between covered stent implantation and TAE tailored to the patient’s lesion characteristics, anatomical location, and clinical manifestations. Although the efficacy and safety of stent-graft implantation for the treatment of HAP have been preliminarily validated, it is worth noting that existing relevant studies are limited by small sample sizes and short follow-up periods. The long-term efficacy and safety still need to be further verified.

## Data Availability

The original contributions presented in the study are included in the article/supplementary material, further inquiries can be directed to the corresponding authors.
